# Association of RS708272 (*CETP* Gene Variant) with Lipid Profile Parameters and the Risk of Myocardial Infarction in the White Population of Western Siberia

**DOI:** 10.3390/biom9110739

**Published:** 2019-11-14

**Authors:** Sergey Semaev, Elena Shakhtshneider, Pavel Orlov, Dinara Ivanoshchuk, Sophia Malyutina, Valery Gafarov, Yuliya Ragino, Mikhail Voevoda

**Affiliations:** 1 Institute of Internal and Preventive Medicine – branch of Institute of Cytology and Genetics, Siberian Branch of Russian Academy of Sciences (SB RAS), 630089 Novosibirsk, Russia; sse85@ngs.ru (S.S.);; 2 Federal research center Institute of Cytology and Genetics, SB RAS, 630090 Novosibirsk, Russia

**Keywords:** rs708272, risk of cardiovascular disease, lipid profile, white population, Western Siberia

## Abstract

The *TaqI* B (rs708272) single-nucleotide variant, i.e., the +279 G/A substitution in intron 1 of the *CETP* gene, is actively investigated as a risk factor of lipid metabolism disorders. The aim of this study was to analyze the association of rs708272 with lipid parameters and the risk of myocardial infarction in the population of Western Siberia (Russia). The study population was selected from a sample surveyed within the framework of the Health, Alcohol and Psychosocial Factors In Eastern Europe (HAPIEE) study (9360 participants, >90% white, aged 45–69 years, males: 50%). In total, 3132 randomly selected patients were included. Plasma lipid levels were determined by standard enzymatic assays. Rs708272 was analyzed by RT-PCR via TaqMan single-nucleotide polymorphism (SNP) Genotyping Assays (Thermo Fisher Scientific, USA). The frequencies of rs708272 genotypes AA (homozygote), AG (heterozygote), and GG were 0.21, 0.49, and 0.30, respectively, in this population. Allele A frequency was 0.46. We found an association of allele G with low levels of high-density lipoprotein cholesterol and a high index of atherogenicity in this population (*p* < 0.001 and *p* < 0.001, respectively). Allele G was significantly associated with the risk of myocardial infarction among the male participants (odds ratio 1.96, 95% confidence interval 1.208–3.178, *p* = 0.008) and in the study population (odds ratio 1.465, 95% confidence interval 1.028–2.087, *p* = 0.036). Thus, rs708272 is associated with myocardial infarction in the white population of Western Siberia (Russia).

## 1. Introduction

Cardiovascular diseases (CVDs) are the leading cause of death in developed countries [1]. The lipid metabolism disorder that underlies the atherosclerotic process is one of the main risk factors of CVDs [2]. Genetic markers of predisposition to lipid metabolism disorders can be used for the early diagnosis and for the development of programs for primary prevention of CVD.

There is a correlation between the level of high-density lipoprotein cholesterol (HDL-C) and the risk of CVD; this correlation is in part explainable by the participation of HDL in reverse cholesterol transport [3]. The latter is a process of transfer of cholesterol esters via HDL particles into the liver, with subsequent cholesterol oxidation to bile acids. One of the key proteins taking part in this phenomenon is cholesterol ester transport protein (CETP). This is a hydrophobic glycoprotein regulating the reverse cholesterol transport from peripheral tissues to the liver and participating in the exchange of cholesterol esters and triacylglycerides between various classes of lipoproteins. A change in the activity and concentration of CETP causes disturbances in reverse cholesterol transport as well as transformation of atherosclerosis manifestations [4].

The *CETP* gene is located in the chromosomal region 16q21, consists of 16 exons, and produces a hydrophobic glycoprotein 476-amino acids long (https://www.ncbi.nlm.nih.gov/gene/1071). Several polymorphisms of *CETP* are known, including rs5883, rs247616, rs247617, rs1532624, rs1800775, rs3764261, and rs9989419, which lead to changes in the amino acid sequence and/or function of the protein [5,6,7].

*TaqI B* (rs708272) is the best-studied polymorphism of this gene and is a G > A substitution at position +279 of intron 1, thereby giving rise to alleles B1 and B2. Allele B1 affects the size of the CETP protein, its function, and the HDL-C level. Allele B2 results in a lower molecular weight CETP and in higher HDL-C concentration and—according to some studies—correlates with a lower risk of ischemic heart disease [8,9,10,11]. Other research indicates sex-specific dependence of the associations of the *TaqI* B polymorphism as well as dependence on the body mass index (BMI), alcohol consumption, and insulin level [12,13]. These effects may be caused by other polymorphisms linked to *TaqI* B.

Our aim was to analyze the association of rs708272 with lipid profile parameters and with the risk of myocardial infarction in the population of Western Siberia (Russia).

## 2. Materials and Methods

A cross-sectional epidemiological study of the adult population was conducted in Novosibirsk (Western Siberia, Russia). From the Novosibirsk residents surveyed during 2007–2008 within the framework of the Health, Alcohol and Psychosocial Factors In Eastern Europe (HAPIEE) study [14], we compiled the main representative sample (9360 subjects, 45–69 years old [mean ± standard error: 53.8 ± 7.0 years], >90% whites) by means of a table of random numbers. The study protocol was approved by the Ethics Committee of the Institute of Internal and Preventive Medicine—a branch of the Institute of Cytology and Genetics, the Siberian Branch of the Russian Academy of Sciences (approval #7 of 22 June 2008). Each participant provided informed consent to be examined and to collection of a blood sample for the study.

The clinical examination program included collection of sociodemographic data, clinical examination, a standard questionnaire on tobacco smoking, anthropometric measurements (height, body weight, and waist circumference), measurement of arterial blood pressure (BP), and assays of biochemical indicators in blood serum (total cholesterol [TC], HDL-C, triglycerides [TGs], and fasting glucose). Blood collection from the median cubital vein was performed in the morning after a 12 h overnight fast. Parameters of the blood lipid profile (TC, TGs, HDL-C, and low-density lipoprotein cholesterol [LDL-C]) were measured by enzymatic assays using standard kits (Biocon Fluitest; Lichtenfels, Germany) on a Labsystem FP-901 biochemical analyzer (Helsinki, Finland). An index of atherogenicity (IA) was calculated via the following formula: IA = (TC − HDL-C)/HDL-C.

For molecular genetic analysis, from the main representative sample, we selected 3132 subjects (hereafter: study population or study subjects) by the random-number method. To isolate DNA from the blood, phenol–chloroform extraction was performed [15]. Genotyping of rs708272 was conducted by means of TaqMan single-nucleotide polymorphism (SNP) Genotyping Assays (Thermo Fisher Scientific, Foster City, CA, USA) and the BioMaster HS-qPCR HI-ROX Kit (Biolabmix, Novosibirsk, Russia) on a StepOnePlus real-time PCR System (Thermo Fisher Scientific). The laboratory personnel performing the genotyping were blinded to the physical and clinical examination data.

Significance of the differences in allele frequencies among the subgroups under study and conformance to the Hardy–Weinberg equilibrium were determined by the χ^2^ test. Evaluation of differences in mean continuous variables among various genotypes was carried out after adjustment for sex, age, and BMI via the generalized linear model (GLM) in the SPSS software for Windows.

## 3. Results

The characteristics of the examined subjects are presented in Table 1. Males constituted 48% and females 52% of the study population. The prevalence of arterial hypertension (>140/90 mmHg) was 42.3%, that of type 2 diabetes mellitus 7.9%, and that of dyslipidemia (TC > 200 mg/dL or 5.2 mM) 82.8%.

For rs708272 in the study population, the distribution of genotype frequencies complied with the Hardy–Weinberg equilibrium (χ^2^ = 1.36). The frequency of allele A of *CETP* rs708272 in the white population of Western Siberia (Table 2) was consistent with its frequency in the white populations of Eastern and Western Europe (according to gnomAD Genomes European data: G frequency = 0.5541, A frequency = 0.4459 (http://www.ncbi.nlm.nih.gov).

After the analysis of associations of rs708272 with parameters of the blood lipid profile (TC, HDL-C, LDL-C, TGs, and the index of atherogenicity), the differences in mean HDL-C levels among the genotypes were found to be statistically significant in the study population (*p* < 0.001), among males (*p* = 0.006) and among females (*p* < 0.001). The lowest concentrations of HDL-C were registered in allele G carriers (Table 3). In all subgroups, the highest mean index of atherogenicity was noted in genotype GG carriers, and this difference was statistically significant both among females and in the study population (*p* < 0.001 and *p* < 0.001, respectively).

In the generalized linear model of the subgroups being studied, we did not detect statistically significant associations of rs708272 with TC, LDL-C, TGs, fasting glucose, BMI, systolic BP, diastolic BP, or heart rate (Table 3).

Thus, we uncovered statistically significant associations of rs708272 genotypes with HDL-C and with the index of atherogenicity in the white population of Western Siberia.

During a 10-year period (2007–2017), in the main representative sample (N = 9360), investigators collected data on new cases of myocardial infarction at the Novosibirsk City Registry of Myocardial Infarctions [16]. During this observation period, 509 new cases of myocardial infraction were registered. In the study population (*n* = 3132), which was genotyped for rs708272, there were 137 cases of myocardial infarction during the 2007–2017 period. In the white population of Western Siberia, a significant association of rs708272 with myocardial infarction was confirmed among males (Table 4). The carriage of allele G was found to be a risk factor of myocardial infarction.

It should be noted that the presence of allele G in various populations has been linked with changes in the activity of the CETP protein, thus leading to lower HDL-C levels, which can raise the risk of CVDs [6,8,9].

## 4. Discussion

The individual risk of CVDs depends both on genetic factors and on lifestyle factors. In this study, we present the results of an examination of a white population regarding a possible association of rs708272 (in the *CETP* gene) with clinical and biochemical parameters. We found that some changes in genomic DNA sequence are independent risk factors of myocardial infarction, in agreement with the findings of other studies [17,18,19,20,21,22,23,24]. The severity of the prognosis depends on the presence of a certain allele or genotype, according to our results.

In our study, rs708272 allele G turned out to be statistically significantly associated with the mean levels of HDL-C in the white population of Western Siberia as well as with the index of atherogenicity among the females in the study population (*p* < 0.001); a similar association for the index of atherogenicity was noted among males but did not reach significance. Previously, we reported a statistically significant association between the genotypes of polymorphism *TaqI* B (rs708272) and HDL-C in subgroups defined by different mean TC concentrations [25]. A higher HDL-C level correlated with the B2B2 (GG) genotype in the group of patients with hypercholesterolemia in that study. This finding underscores the importance of not only population-level studies but also research on subjects with specific phenotypes of the lipid profile in order to determine the contribution of genetic factors to the predisposition to common diseases. These observations are consistent with the results of several studies [6,8,9]. For instance, Gilberto Vargas-Alarcon and coworkers demonstrated an association of allele G with a lowered HDL-C concentration and a higher risk of acute coronary syndrome (odds ratio = 1.45, *p* = 0.036) [8].

In 2016, Guo and colleagues conducted a meta-analysis and revealed a correlation of genotype AA with a higher HDL-C level (as compared to genotype GG carriers; *p* < 0.001) as well as with an elevated risk of cardiovascular events in allele G carriers (odds ratio = 1.15, *p* < 0.001) among subjects from Asian and white populations [9]. Previously, a similar association was found for a Chinese population in a meta-analysis by Li Y. et al. in 2013 [26].

For the Russian population, the evaluation of the usefulness of myocardial infarction genetic markers (identified in genome-wide association studies) has yielded inconsistent results [27]. Out of nine single-nucleotide variants (SNVs) associated with myocardial infarction in other white populations, only five SNVs showed an association with myocardial infarction in the white population of Russia [27]. The research on the role of SNVs in the development of pathologies in various populations indicates their clinical value and allows investigators to select the most reliable markers manifesting an association not only with the main pathological phenotype but also with its risk factors.

We uncovered an independent statistically significant contribution of the rs708272 SNV to the risk of myocardial infarction among males in the white population of Western Siberia. A sex-specific association of rs708272 with the blood lipid level but not the risk of CVD was demonstrated by Cai G. and coworkers in the Asian Han population in 2018 [28]. In our study, the absence of a statistically significant association of rs708272 with the risk of myocardial infarction in females is probably related to the development of ischemic heart disease in females at a later age as compared to males [29,30,31].

The present study has some limitations. We examined only rs708272 and traditional cardiovascular risk factors and thus could not rule out the influence of other factors, including lifestyle factors, that may affect the results of observational studies.

The research into genetic risk factors of CVDs is important for the development of primary-prevention programs, considering that it is possible to determine genetic variation before the first clinical manifestations of the disease. In addition, the information about genetic risk factors of the disease may help to optimize the clinical management of the patients.

## Figures and Tables

**Table 1:** Characteristics of study subjects from the population of Western Siberia (Russia).

	Males	Females	Both Sexes
Number of subjects	1503	1628	3132
Age, years	56.6 ± 0.2	56.5 ± 0.2	56.5 ± 0.1
TC, mg/dL	235.5 ± 1.4	253.4 ± 1.5	244.8 ± 1.1
HDL-C, mg/dL	57.9 ± 0.4	60.8 ± 0.5	59.4 ± 0.3
LDL-C, mg/dL	115.9 ± 1.2	127.6 ± 1.3	122 ± 0.9
TGs, mg/dL	136.1 ± 2	143.1 ± 2.1	139.8 ± 1.5
Index of atherogenicity	2.8 ± 0.03	2.9 ± 0.03	2.8 ± 0.03
Fasting glucose, mmol/L	5.7 ± 0.06	5.7 ± 0.06	5.7 ± 0.04
Body mass index, kg/m^2^	26.5 ± 0.1	29.7 ± 0.1	28.1 ± 0.1
Waist circumference, cm	94.8 ± 0.3	92.3 ± 0.4	93.5 ± 0.2
Systolic blood pressure, mmHg	143.1 ± 0.6	143.6 ± 0.6	143.4 ± 0.4
Diastolic blood pressure, mmHg	90 ± 0.3	89.8 ± 0.3	89.9 ± 0.2
Heart rate, bpm	71.5 ± 0.3	71.7 ± 0.3	71.6 ± 0.2

* Continuous variables are presented as mean ± standard error. TGs, triglycerides; TC, total cholesterol; HDL-C, high-density lipoprotein cholesterol; LDL-C, low-density lipoprotein cholesterol.

**Table 2:** Frequencies of alleles and genotypes of rs708272.

	Males	Females	Both Sexes
	%	%	%
Genotypes			
AA	0.22 *n* = 334	0.21 *n* = 336	0.21 *n* = 670
AG	0.47 *n* = 707	0.50 *n* = 815	0.49 *n* = 1522
GG	0.31 *n* = 463	0.29 *n* = 477	0.30 *n* = 940
Alleles			
A	0.4571	0.4566	0.4568
G	0.5428	0.5433	0.5431

*n*: the number of individuals (3132 total).

**Table 3:** Analysis of rs708272 association with parameters of the blood lipid profile and with results of the clinical examination in the white population of Western Siberia (*n* = 3132).

Sex	Genotype	TC, mg/dL	HDL-C, mg/dL	LDL-C, mg/dL	TGs, mg/dL	Index of Atherogenicity	Fasting Glucose, mM	BMI, kg/m^2^	Systolic BP, mmHg	Diastolic BP, mmHg	Heart Rate, bpm
Males	AA	238.5 ± 3	59.6 ± 0.8	115.3 ± 2.5	140.5 ± 4.7	2.72 ± 0.07	5.85 ± 0.14	26.4 ± 0.2	144 ± 1.3	90.2 ± 0.8	71.8 ± 0.7
	AG	236.3 ± 2.1	58.2 ± 0.6	116.8 ± 1.7	135.2 ± 2.8	2.78 ± 0.05	5.57 ± 0.07	26.5 ± 0.2	143.5 ± 0.9	90.5 ± 0.5	71.6 ± 0.5
	GG	232.1 ± 2.4	56.2 ± 0.7	114.9 ± 2.1	134.5 ± 3.7	2.86 ± 0.07	5.79 ± 0.11	26.4 ± 0.2	141.8 ± 1.1	89.1 ± 0.6	71.2 ± 0.6
*p*		0.317	0.006 *	0.578	0.631	0.226	0.06	0.948	0.428	0.265	0.734
Females	AA	251.5 ± 3.3	65.3 ± 1.7	123.7 ± 3.1	137.7 ± 4.6	2.62 ± 0.07	5.69 ± 0.12	29.6 ± 0.3	144.6 ± 1.4	90.3 ± 0.7	71.2 ± 0.6
	AG	254.9 ± 2.3	59.9 ± 0.5	128.7 ± 1.9	146.3 ± 3.1	2.94 ± 0.05	5.75 ± 0.09	29.8 ± 0.9	143 ± 0.9	89.5 ± 0.5	71.9 ± 0.4
	GG	252.3 ± 2.7	59.1 ± 0.6	128.3 ± 2.3	141.5 ± 3.6	2.99 ± 0.07	5.59 ± 0.09	29.6 ± 0.3	144.1 ± 1.1	90 ± 0.6	71.8 ± 0.5
*p*		0.426	<0.001 *	0.314	0.152	<0.001 *	0.452	0.838	0.354	0.461	0.532
Both sexes	AA	245 ± 2.2	62.5 ± 1	119.5 ± 2	139.1 ± 3.3	2.67 ± 0.05	5.77 ± 0.09	28 ± 0.2	144.3 ± 1	90.3 ± 0.5	71.5 ± 0.4
	AG	246.2 ± 1.6	59.1 ± 0.4	123.2 ± 1.3	141.1 ± 2.1	2.87 ± 0.04	5.67 ± 0.06	28.3 ± 0.1	143.2 ± 0.6	90 ± 0.3	71.7 ± 0.3
	GG	242.4 ± 1.8	57.7 ± 0.5	121.7 ± 1.6	138 ± 2.6	2.93 ± 0.05	5.68 ± 0.07	28 ± 0.2	143 ± 0.8	89.6 ± 0.4	71.5 ± 0.4
*p*		0.426	<0.001 *	0.314	0.152	<0.001 *	0.452	0.838	0.354	0.461	0.532

The data are presented as mean ± standard error. BMI, body mass index; BP, blood pressure. * Statistically significant.

**Table 4:** The association of rs708272 with myocardial infarction in the white population of Western Siberia (*n* = 3132).

Sex	Genotype	Subgroup		Myocardial Infarction Cases		OR (95% CI)	*p*
		*n*	%	*n*	%		
Males	AA	323	22.5	11	15.7	0.641 (0.333–1.235)	0.238
	AG	680	47.4	27	38.6	0.696 (0.426–1.139)	0.177
	GG	431	30.1	32	45.7	1.960 (1.208–3.178)	0.008 *
Females	AA	323	20.7	13	19.4	0.923 (0.497–1.711)	0.878
	AG	781	50	34	50.7	1.029 (0.631–1.678)	1.000
	GG	457	29.3	20	29.9	1.028 (0.602–1.754)	0.892
Both sexes	AA	646	21.6	24	17.5	0.770 (0.491–1.206)	0.287
	AG	1461	48.9	61	44.5	0.838 (0.594–1.183)	0.337
	GG	880	29.5	52	38.0	1.465 (1.028–2.087)	0.036 *

* Statistically significant. OR: odds ratio, CI: confidence interval.

## Abbreviations

CVD	cardiovascular disease
HDL-C	high-density lipoprotein cholesterol
LDL-C	low-density lipoprotein cholesterol
PCR	polymerase chain reaction
SNV	single-nucleotide variant
TC	total cholesterol
TGs	triglycerides
